# MALT lymphoma: epidemiology, clinical diagnosis and treatment

**DOI:** 10.25122/jml-2018-0035

**Published:** 2018

**Authors:** Petruta Violeta Filip, Denisa Cuciureanu, Laura Sorina Diaconu, Ana Maria Vladareanu, Corina Silvia Pop

**Affiliations:** 1. “Carol Davila” University of Medicine and Pharmacy, Bucharest; 2. Department of Internal Medicine II and Gastroenterology, Emergency University Hospital, Bucharest

**Keywords:** gastric lymphoma, Helicobacter pylori, mucosa-associated lymphoid tissue lymphoma, diffuse large B-cell lymphoma, HP – Helicobacter pylori, PGL – Primary gastric lymphoma, NHL – Non-Hodgkin lymphomas, MALT – Mucosa-associated lymphoid tissue, DLBCL – Diffuse large B-cell lymphoma, HIV – Human immunodeficiency virus, EBV – Epstein-Barr Virus, CagA protein – Cytotoxin-associated gene A protein, NF-κB – Nuclear factor kappa B, TLR4 – Toll-like receptor 4, CT – Computer tomography, EUS – Endoscopic ultrasonography, PET – Positron emission tomography, PPI – Proton-pump inhibitor, BID – Twice daily, QD – Once daily, TID – Three times daily, QID – Four times daily

## Abstract

Primary gastric lymphoma (PGL) represents a rare pathology, which can be easily misdiagnosed because of unspecific symptoms of the digestive tract. Histologically, PGL can vary from indolent marginal zone B-cell lymphoma of the mucosa-associated lymphoid tissue (MALT) to aggressive diffuse large B-cell lymphoma (DLBCL). During the years, clinical trials revealed the important role of Helicobacter pylori (H. pylori) in the pathogenesis of gastric MALT lymphoma. Infection with Helicobacter pylori is an influential promoter of gastric lymphomagenesis initiation. Long-term studies revealed that eradication therapy could regress gastric lymphomas.

## Introduction

Primary gastric lymphoma (PGL) is the most common extranodal site of non-Hodgkin lymphoma and represents 30% to 40% of all extranodal lymphomas [1]. It also represents 4% to 20% of all non-Hodgkin lymphomas (NHL) and approximately 5% of primary gastric neoplasms [2]. The frequent histological subtypes of PGL are marginal zone B-cell lymphoma of the mucosa-associated lymphoid tissue (MALT) and diffuse large B-cell lymphoma (DLBCL) [3].

It is important to mention that MALT lymphomas are low-grade lesions and DLBCL gastric lymphomas are high-grade and more common than the first one [3]. Treatment is different for these two pathologies.

The incidence of developing PGL is 2–3 times higher in males than in females [4]. MALT lymphoma has the highest incidence between the age of 50 and 60 years [5], but it was observed that the incidence increased significantly in patients older than 40, in conformity with the data collected in the First Affiliated Hospital of Dalian Medical University in a retrospective study from January 1999 to December 2010 [6].

Diffuse large B-cell lymphomas (DLBCL) are composed of large B-cells, this pathology being developed de novo, but are also described cases evolving from transformed MALT lymphomas [7].

## Pathogenesis

This type of lymphoma is typically a low-grade neoplasia, characterized by a dense lymphoid infiltration that invades and destroys gastric glands and results in the so-called “lymphoepithelial lesion” which is pathognomonic for lymphoma [8].

MALT lymphomas do not have a specific antigenic profile, the B-cells sharing the immunophenotype with marginal zone B-cells present in the spleen, Peyer’s patches and lymph nodes, so gastric lymphoma is CD20+, CD5+; CD10-, CD23- and cyclin D1- [9].

Gastric MALT lymphomas are strongly associated with *Helicobacter pylori* infection. This pathogen is the most common infectious agent related to worldwide cancers (5.5% of total cancers) [10]. This bacteria was discovered in 1982; its prevalence depends on geographic regions, socio-economic status, education level, age, living environment and occupation.

In the last years, it has been discovered that this kind of infection also plays a role in the pathology of DLBCL gastric lymphomas [11]. Usually, gastric MALT lymphoma is a low-grade lesion, but it can turn into a high-grade lymphoma. Patients who have H. pylori gastritis are at risk to develop gastric MALT lymphomas. This pathology has a low incidence, and the development of neoplasia requires some conditions [9]. Experimental studies showed that only 1 of the 13 different tested H. pylori strains was able to stimulate B-cell proliferation and to produce IL-2 by T-cells [12]. Escherichia coli and Campylobacter jejuni were also tested, two Gram-negative intestinal bacteria which share different antigens with HP, and they failed to induce B-cell proliferation in culture, so HP strains have a specific role [12]. It was discovered that MALT lymphoma expresses high levels of a ligand (APRIL), a novel cytokine which is crucial in sustaining B-cell proliferation [13]. Also, H. pylori and H. pylori-specific T cells stimulate the macrophages to produce APRIL [14]. In addition, H. pylori can translocate the CagA protein directly into B-cells resulting in extracellular signal-regulated kinase activation and Bcl-2 expression up-regulation, leading to apoptosis inhibition [15]. Recent studies showed that CagA positive strains are more frequent in DBCL than in low-grade MALT lymphoma [16]. Patients infected with this type of H. pylori strains are responders to eradication therapy [15]. During HP infection, normal B cells are transformed into malignant clone via three chromosomal translocations – t(11;18) (q21;q21), t(1;14)(p22;q32), and t(14;18)(q32;q21), which produces activation of nuclear factor kappa B (NF-κB), which plays a role in immunity, inflammation, and apoptosis [17–20]. Studies show that t(11;18)(q21;q21) was found to be more prevalent in patients with CagA-positive H. pylori strains which determine MALT lymphoma [21].

Genetics play an essential role in the development of gastric lymphoma. Patients with MALT lymphomas have a high prevalence of HLA-DQA1*0103, HLA-DQB1*0601 and R702W mutation in the NOD2/CARD15 gene [22, 23]. Those with low-grade lymphoma are associated with the presence of TNF-857T allele [15]. A significant role in the genetic susceptibility of gastric lymphoma plays a rare allele of the Toll-like receptor 4 (TLR4 Asp299Gly) [24]. Genetics protect the patients against high-grade lymphoma, but not of low-grade, by homozygous haplotypes for the rare allele G of SNP3 (rs12969413) [25].

In the last few years, it has been observed that other environmental factors increase the incidence of HP infection and carcinogenesis. Obesity is one of them; the latest clinical epidemiological trials have revealed that metabolic syndrome and obesity result in an increased incidence of HP infections [10,26]. Insulin resistance and hyperglycemia are co-factor risks in HP-induced gastric carcinogenesis [27]. Also, high dietary salt could stimulate the expression of CagA gene in those who are infected with HP [28].

Pathogenesis of primary gastric lymphoma could be associated with another risk factors like hepatitis B virus, human immunodeficiency virus (HIV), Epstein-Barr virus and human T- cell lymphotropic virus type 1 [4].

**MALT lymphoma clinical manifestation and endoscopic appearance**

Gastric MALT lymphoma is often an indolent, multifocal disease and because of that, it has a high rate of relapse after surgical excision. In 10% of cases, it can have synchronous involvement of intestinal and extraintestinal sites [29].

Clinical presentation may vary from symptoms such as nausea, vomiting, dyspepsia and epigastric pain to massive hemorrhage, chronic gastric bleeding with iron-deficiency anemia, pyloric stenosis and weight loss. In rare cases, fever or night sweats can be present (typical manifestation of B-cell lymphoma), or it can debut with perforation when there is a massive infiltration of the gastric wall [29, 30].

Gastric MALT lymphoma can involve any part of the organ, but more frequently it affects the antrum.

Endoscopic appearance can vary from irregularly shaped superficial erosions, shallow ulcers to enlarged rugal folds, intra-gastric nodularities or thickened gastric walls (Table 1) [18].

The diagnosis can be established based on the endoscopic appearance, although sometimes the mucosa may have normal appearance while the biopsy is the “gold standard”. Sometimes, we can miss the diagnosis by conventional pinch biopsies because gastric MALT can infiltrate only the submucosa [31].

In the last years, magnifying endoscopy has improved endoscopic diagnosis of gastric lymphoma by detection of destructed gastric pits, irregular pit size or distribution [18].

It is necessary to perform a stomach mapping. The endoscopist must take specimens from the antrum and the corpus for the urease test. If the examined segments are unsuspicious, a biopsy must be performed from each quadrant of the fundus, the corpus and the antrum and ten biopsies if they are suspicious, in order to request a histological work-up.

**Staging of gastric MALT lymphoma**

Staging of this pathology is mandatory because morbidity and mortality are high, even though usually this type of lymphoma is low-graded. These procedures include a complete physical examination, laboratory tests (blood count, renal and liver test, lactate dehydrogenase and β2-microglobulin), imaging tests and also a bone marrow biopsy. Almost 10% of low-grade MALT lymphoma are in advanced stages (III-IV) and require anti-neoplastic therapy [22]. Advanced stages of the disease involve dissemination to the lymph nodes, lungs, liver and bone marrow. The imaging tests used for staging are computed tomography (CT) of the chest, abdomen and pelvis, endoscopic ultrasonography (EUS) and positron emission tomography (PET). The endoscopic ultrasonography is necessary to determine the infiltration of the gastric wall, the involvement of the regional lymph nodes and it is also used in the remission follow-up [32–34].

**Table 1:** Endoscopic classification of gastric MALT-lymphoma. Data modified after Zullo A et al. [16]

Lymphoma type	Endoscopic presentation
Normal/hyperaemic mucosa	Normal mucosa/hyperaemic modification
Petechial haemorrhages	Presence of mucosal petechial haemorrhages
Hypertrophic	Nodular pattern; large or giant folds
Exophytic	Irregular mucosa - tumor-like appearance or polypoid mass
Ulcerative	Multiple erosions/single or multiple ulcerations
Mixed	A combination of more than one pattern

In gastric lymphomas, three different EUS patterns can be found. This procedure is necessary to differentiate between lymphoma and gastric carcinomas. EUS is more useful than computed tomography in evaluating the remission of the disease because it can evaluate the extraluminal extent of the disease or the extension in the lymph nodes [18, 35, 36].

Conventional X-ray does not play an important role in diagnosing lymphoma, but it can appreciate the extent of the disease. It can reveal a normal aspect or thicker gastric mucosal folds, ulceration or linitis plastica.

Computer tomography determines the involvement of the wall, including submucosal spreading and also the spreading to the lymph nodes [35]. The aspect may vary from rugged mucosa to polypoidal mass or ulcerations.

**Figure 1:**
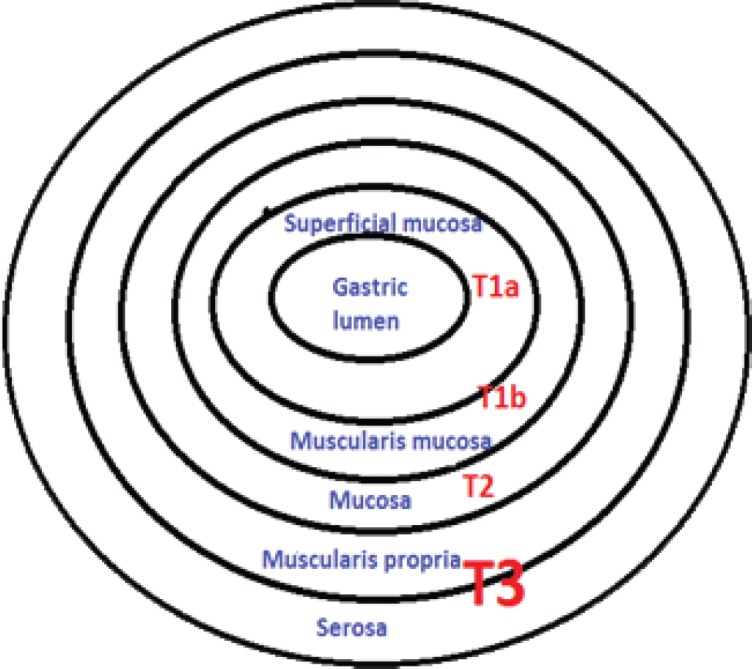
Endoscopic Ultrasonography Staging of Gastric MALT Lymphoma[37]

**Endoscopic ultrasound staging of gastric lymphoma** (Figure 1) [37]: stage T1a – the superficial mucosa is affected and the first layer is hyperechoic; stage T1b – the lesion is extended from deeper mucosa to muscularis mucosa; stage T2 is represented by lesions in the submucosa (hyperechoic layer on EUS); stage T3 affects muscularis propria and serosa.

**Ann Arbor Classification of Gastric Lymphoma (Modified by Musshoff)** [37,38]: IE – lesions restricted to the stomach (no lymph node involvement) IE1 – limited to the mucosa and submucosa IE2 – extension to the submucosa IIE – extension to the lymph nodes IIE1 – extension to the perigastric lymph nodes IIE2 – involvement of the somach and extension to the sub-diaphragmatic lymph nodes IIIE – involvement of the gastrointestinal tract or/and extension to the lymph nodes on both sides of the diaphragm IVE – extension to the extra-gastrointestinal tissues or organs.

**Prognostic factors**

Several prognostic factors can influence the survival of patients with primary gastric lymphoma. The most important prognostic factor for PGL is the international prognosis index (IPI). This index includes age (> 60 years), poor performance status, elevated LDH and advanced stage [4].

In September 2017, a new MALT lymphoma prognostic index (MALT-IPI) was published [4]. Thieblemont et al. claimed that age > 70 years, elevated LDH and Ann Arbor stage III or IV are the most important prognostic factors, being an important tool for MALT lymphoma patients with poor outcomes [4]. The literature reported that t(11;18) is associated with resistance to antibiotic therapy that gives a poor prognosis [4].

**Therapeutic management of MALT lymphoma**

***Therapy of infected patients with* Helicobacter pylori**

The discovery of HP role in the pathogenesis of gastric lymphomas has radically changed the prognosis of these patients, with an increased survival rate just by eradicating this pathogen. Current gastroenterology and oncology international guidelines have established that the first line therapy in the early stages is the eradication of HP, while in those with advanced stages, adjuvant anti-tumor therapy is needed.

Eradication therapy referred to as “first-line” therapy, must be carefully selected for each patient because any therapy can guarantee the cure of *Helicobacter pylori* infection in 100% of cases if there is no known resistance. Typically, this infection can be treated by combining a proton pump inhibitor (PPI) with 2–3 antibiotics concomitantly or sequentially for a period of 7 to 14 days according to the American College of Gastroenterology guidelines [34]. In the past, the treatment recommendation was a combination of a PPI, Clarithromycin and Amoxicillin (Clarithromycin-based triple therapy) or Metronidazole for those with an allergy to Amoxicillin, for a period of 14 days (Table 2). Clarithromycin resistance became an important issue in different parts of the world – where the success rate of eradication is under 80% and Clarithromycin therapy is now recommended to those with resistance <15% and no previous exposure to macrolides [39]. Data research concluded that PPI, Clarithromycin and Amoxicillin or Metronidazole for 14 days should be the first-line treatment only where resistance is low [34].

In Europe, the Maastricht IV guidelines (2016) recommended that first-line and second-line therapies should be clarithromycin-based regimens and bismuth-based quadruple therapy (PPI, Bismuth, Tetracycline and a Nitroimidazole for 10–14 days), respectively. [40]. If those two regimens fail, a third-line therapy with a fluoroquinolone-based regimen (PPI, amoxicillin and levofloxacin) is recommended. Hybrid therapies and sequential therapies are missing from the European recommendations [40].

***The role of probiotics in first-line therapy***

In the last years, probiotics have become an adjuvant therapy for treating *Helicobacter pylori* infections. *Lactobacillus* and *Bifidobacterium* species have an inhibitory effect on *H. pylori*, also reducing the side effects of eradication regimens and improving the compliance [41, 42]. In 2015, Zhang et al. made a systematic review and a meta-analysis that used the results from 45 studies and revealed an improved eradication rate of *Helicobacter pylori* by using probiotics associated to the standard therapy [42]. Meta-analyses that include *Saccharomyces boulardii* associated with the triple eradication regimen also revealed an increased *H. pylori* eradication rate [40, 43]. Studies that include *Lactobacillus reuteri* have shown improvement in gastrointestinal side effects, but regarding eradication rates, data are not conclusive [43].

The management of gastric MALT lymphoma must take into consideration the eradication of *H. pylori* independently of the stage. Those patients who have benefited from eradication therapy must be evaluated with urea breath test at least 6 weeks after eradication and at least 2 weeks after IPP discontinuation.

**Table 2:** Therapeutic regims indicated for Helicobacter pylori eradication [35].

Therapy regim	Drugs	Doses per day	Days of treatment
Clarithromicin triple therapy	PPI+ Clarithromicin + Amoxacillin	40mg BID + 500mg BID+ 1g BID	14
	PPI+ Clarithromicin + Metronidazole*	40mg BID + 500mg BID+ 500mg TID	
Bismuth quadruple therapy	PPI+Bismuth subcitrat/subsalicylate +Tetracycline+Metronidazole	40mg BID+120-300mgQID/300mgQID +500mgQID+500mgTI D	10-14
Concomitant therapy	PPI+Clarithromicyn+Amox acillin+Nitroimidazole	40mg BID +500mg BID+500mgBID	10-14
Sequential therapy	PPI+Amoxacillin, PPI+ Clarithromicyn +Nitroimidazole†	40mg BID+ 1g BID 40mg BID+500mg BID+ 500mg BID	7 7
Hybrid therapy	PPI+Amoxacillin PPI+ Amoxacillin +Clarithromicyn+Nitroimidazole†	40mg BID+1g BID, 40mg BID+1g BID + 500mg BID+500mg BID	7
Levofloxacin Triple	PPI+ Amoxacilin	40mg BID+ 1g	10
Based therapy	Levofloxacin	BID+500mg QD	
Sequential	PPI +Amoxicillin PPI+Levofloxacin+ Nitroimidazole†	40mg BID+ 500mg QD +500mg TID	7
Quadruple/LOAD	PPI+Levofloxacin+Nitazoxanide+Doxycycline	80mgQD+250mgQD+500mgBID+100mgQD	7-10

Legend: PPI-proton pump inhibitor; BID-twice daily; TID-three times daily; QD-once daily; QID-four times daily.

* for those patients with allergy on Penicillin or with previous exposure on Macrolide (Clarithromycin).

† Metronidazole or tinidazole.

The follow-up for MALT lymphoma includes an endoscopic examination which can be performed in 3–6 months after completion of the eradication treatment and gastric mucosal biopsy sampling for H. pylori. The endoscopic examination plays an important role in MALT lymphoma assessment and establishing endoscopic and histological remission. A post-treatment evaluation of histological remission was proposed in 2012 by the Groupe d’ Etude des Lymphomes de l’ Adulte (GELA) [44]: — Complete histological remission (CR) = lamina propria (LP) with absent or scattered plasma cells and small lymphoid cells; absence of lymphoepithelial lesions; normal stroma or empty LP and/or fibrosis. — Probable minimal residual disease (pMRD) = aggregates of lymphoid cells or lymphoid nodules in the LP or muscularis mucosa; and/or submucosa; absent lymphoepithelial lesions; stroma with empty LP and/or fibrosis. — Responding residual disease (rRD) Dense = Lymphoid infiltrate diffuse or nodular extending around glands in the LP; absent or focal lymphoepithelial lesions; stroma with focal empty LP and/or fibrosis. — No change (NC) = Lymphoid infiltrate nodular or dense diffuse; lymphoepithelial lesions – present, “may be absent”; stroma with no changes.

**Surgery**

Cases with multifocal MALT lymphoma require extensive gastrectomy with consequences in the quality of life. It is often necessary when complications such as major hemorrhage, pyloric stenosis or perforation (cases with massive infiltration and chemotherapy) occur [45].

Surgery is no longer a curative first-line treatment for gastric MALT lymphoma; patients with failure to eradication therapy and those with negative *H. pylori* infection should benefit from alternative therapeutic regimens that include radiation therapy or chemotherapy.

**Radiotherapy**

Moderate-dose radiotherapy (30Gy) including stomach and perigastric lymph nodes offers good results with a remission rate of 93–100%. Because of continual innovation, the radiation dose to the normal tissues has decreased and made possible the conformal delivery of radical radiation to the tumor [46, 47].

Over the years, radiotherapy concepts have changed, so it is useful for limited forms of illness, and chemotherapy is required for advanced stages of the disease. The optimum response time is not well defined due to the indolent nature of MALT gastric lymphoma. It should be noted that the side effects of current radiotherapy are not significant because the studies conducted so far included a small number of patients [48].

**Chemotherapy and immunotherapy**

Chemotherapy and Rituximab immunotherapy could be used in all stages of the disease or a combination of both.

Over the time, studies have shown that the combination of Rituximab (anti-CD20 monoclonal antibodies) and oral alkylating agents (Chlorambucil, Cyclophosphamide) or purine nucleoside analogs (Cladribine, Fludarabine) have superior results in the remission of the disease and survival rates [49]. For those patients with massive tumoral masses, histological transformation or unfavorable International Prognostic Index, an aggressive chemotherapy regimen which contains Anthracycline should be used [49].

New molecules take the place of these agents, bendamustine being a good option treatment for a young patient diagnosed with gastric MALT lymphoma. More clinical trials for proteasome inhibitors such as bortezomib are necessary to discover the right dose to prevent the side effects (hematological and polyneuropathy toxicity). In MALT lymphoma, immunomodulators like lenalidomide alone or combined with rituximab can also be used but more clinical trials are necessary [4].

**Gastric MALT lymphoma therapeutic algorithm [37]:**

:Stage IE

HP+→Eradication Therapy→Radiation

HP-→Radiation Chemotherapy or Surgery

Stage IE2/IIE:

HP+→Radiation ± Eradication Therapy → Chemotherapy

HP-→Radiation→Chemotherapy

StageIIE2/IIIE/IVE:

HP±→Chemotherapy ± Eradication Therapy.

## Conclusions

Primary gastric lymphoma remains a rare pathology. Histologically, the majority of PGL are MALT and DLBCL lymphoma. MALT lymphoma is related to Helicobacter pylori infection.

Regression of gastric MALT lymphoma can be done in the early stages of the disease by eradication therapy.

The majority of cases with MALT lymphoma are cured by eradication therapy, but there are cases that need to be treated with rituximab alone or in combination with other drugs.

It is essential to do a proper endoscopic follow-up and monitor the histological regression using the GELA scoring system to prevent the histological transformation and distant spreading.

Even if this pathology is rare, more clinical trials are required to discover new molecules to cure it.

## Conflict of Interest

The authors confirm that there are no conflicts of interest.
